# Supplementary material for: Cell of origin epigenetic priming determines susceptibility to Tet2 mutation
Source: Nat Commun. 2024 May 21;15:4325. doi: 10.1038/s41467-024-48508-6 (PMC11109152; doi:10.1038/s41467-024-48508-6)
Supplement: Supplementary file 1 — Supplementary Information [file 41467_2024_48508_MOESM1_ESM.pdf]

Supplementary Information

Cell of origin epigenetic priming determines susceptibility to Tet2 mutation.

Schiroli et al.

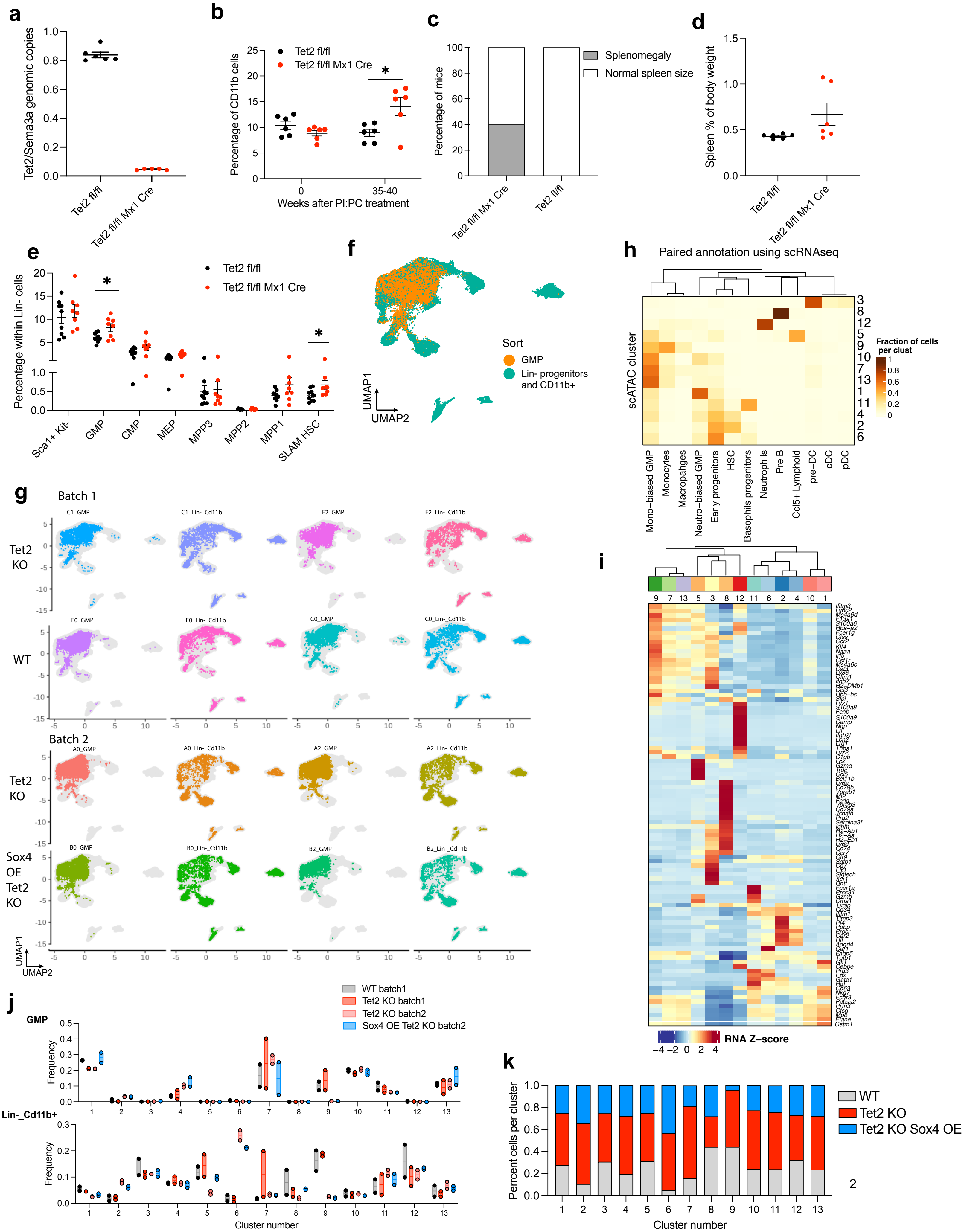
a
Tet2/Sema3a genomic copies
Tet2 fl/fl
Tet2 fl/fl Mx1 Cre
b
Tet2 fl/fl
Tet2 fl/fl Mx1 Cre
Percentage of CD11b cells
Weeks after PI:PC treatment
0
35-40
c
Splenomegaly
Normal spleen size
Percentage of mice
Tet2 fl/fl Mx1 Cre
Tet2 fl/fl
d
Spleen % of body weight
Tet2 fl/fl
Tet2 fl/fl Mx1 Cre
e
Tet2 fl/fl
Tet2 fl/fl Mx1 Cre
Percentage within Lin- cells
Sca1+ Kit-
GMP
CMP
MEP
MPP3
MPP2
MPP1
SLAM HSC
f
Sort
GMP
Lin- progenitors and CD11b+
UMAP1
UMAP2
h
Paired annotation using scRNAseq
scATAC cluster
Fraction of cells per clust
Mono–biased GMP
Monocytes
Macropahges
Neutro–biased GMP
Early progenitors
HSC
Basophils progenitors
Neutrophils
Pre B
Ccl5+ Lymphoid
pre–DC
cDC
pDC
g
Batch 1
C1_GMP
C1_Lin-_Cd11b
E2_GMP
E2_Lin-_Cd11b
Tet2 KO
E0_GMP
E0_Lin-_Cd11b
C0_GMP
C0_Lin-_Cd11b
WT
Batch 2
A0_GMP
A0_Lin-_Cd11b
A2_GMP
A2_Lin-_Cd11b
Tet2 KO
B0_GMP
B0_Lin-_Cd11b
B2_GMP
B2_Lin-_Cd11b
Sox4 OE Tet2 KO
UMAP1
UMAP2
i
RNA Z–score
j
WT batch1
Tet2 KO batch1
Tet2 KO batch2
Sox4 OE Tet2 KO batch2
GMP
Lin-_Cd11b+
Frequency
Cluster number
k
WT
Tet2 KO
Tet2 KO Sox4 OE
Percent cells per cluster
Cluster number

Supplementary Figure 1. *Tet2* KO phenotypic characterization and paired sc-RNA/sc-ATAC dataset annotation.

a) Quantification of Cre activity in the PB of pI:pC treated mice 25 weeks after transplant. Successful *Tet2* deletion was measured by ddPCR comparing the number of copies of *Tet2* and *Sema3a* (used as gDNA normalizer). b) Percentage of CD11b+ myeloid cells in PB before and after *Tet2* deletion by pI:pC treatment. *Tet2*$^{fl/fl}$ n=6; *Tet2*$^{fl/fl}$ Mx1 Cre n=6; Unpaired t-test, p<0.05 c) Percentage of mice showing splenic abnormalities stratified by genotype at terminal endpoint of 35-40 weeks after mutation induction *Tet2*$^{fl/fl}$ n=6, *Tet2*$^{fl/fl}$ Mx1 Cre n=6. d) Data from Supplementary Fig.1c showing spleen weight represented as percentage of body weight. e) percentage of the different BM primitive populations gated within Lin- progenitors. *Tet2*$^{fl/fl}$ n=9; *Tet2*$^{fl/fl}$ Mx1 Cre n=8; Unpaired t-test, p<0.05. f) UMAP plots highlighting distribution of sorted GMP or Lin-/Cd11b populations. g) UMAP plots showing cell distribution for each single mouse and sample analyzed (sorted GMP vs Lin-_Cd11b). Experimental batch of is indicated. Mice belonging to the *Sox4* OE *Tet2* KO group will be discussed in Figure 5 but added here for comparison. h) Heatmap showing the correlation between unbiased clustering using scATACseq data (cluster number is indicated) and unbiased clustering using paired scRNAseq data. scRNAseq data were annotated using key maker gene signatures for each population, as described[35,36]. i) Heatmap representing exemplificative RNA markers utilized for scATAC seq cluster annotation and their RNA expression level. Examples of key RNA markers include: *Ifitm3*, *Txnip*, *Hlf*, *Adgrl4*, *Procr* (HSC and progenitors), *Vpreb3*, *Cd79a/b*, *Fcrla*, *Ccl5* (lymphoid lineage), *Ly86*, *Klf4*, *Irf8*, *Ccr2*, *F13a1*, *Mpo*, *C1qb* (monocytic/mac lineage), *Cebpe*, *Gstm1*, *FcnB*, *S100a8/9*, *Elane*, *Lrg1* (granulocytic lineage), *Cd74*, *Itgb7*, *Naaa*, *Cst3*, *Siglech*, *Cd7*, *Ccr7* (DC lineage), *Prss34* (basophilic lineage). j) Frequency of cells belonging to each cluster for each single mouse and sample analyzed (sorted GMP vs Lin-_Cd11b, same as Supplementary Fig.1g). Experimental batch is indicated, as batch-related differences in cluster abundance are detected (likely related to the sorting procedure). k) Frequency of cells belonging to each cluster divided by genotype. All mice and samples analyzed are considered. Source data are provided as a Source Data file.

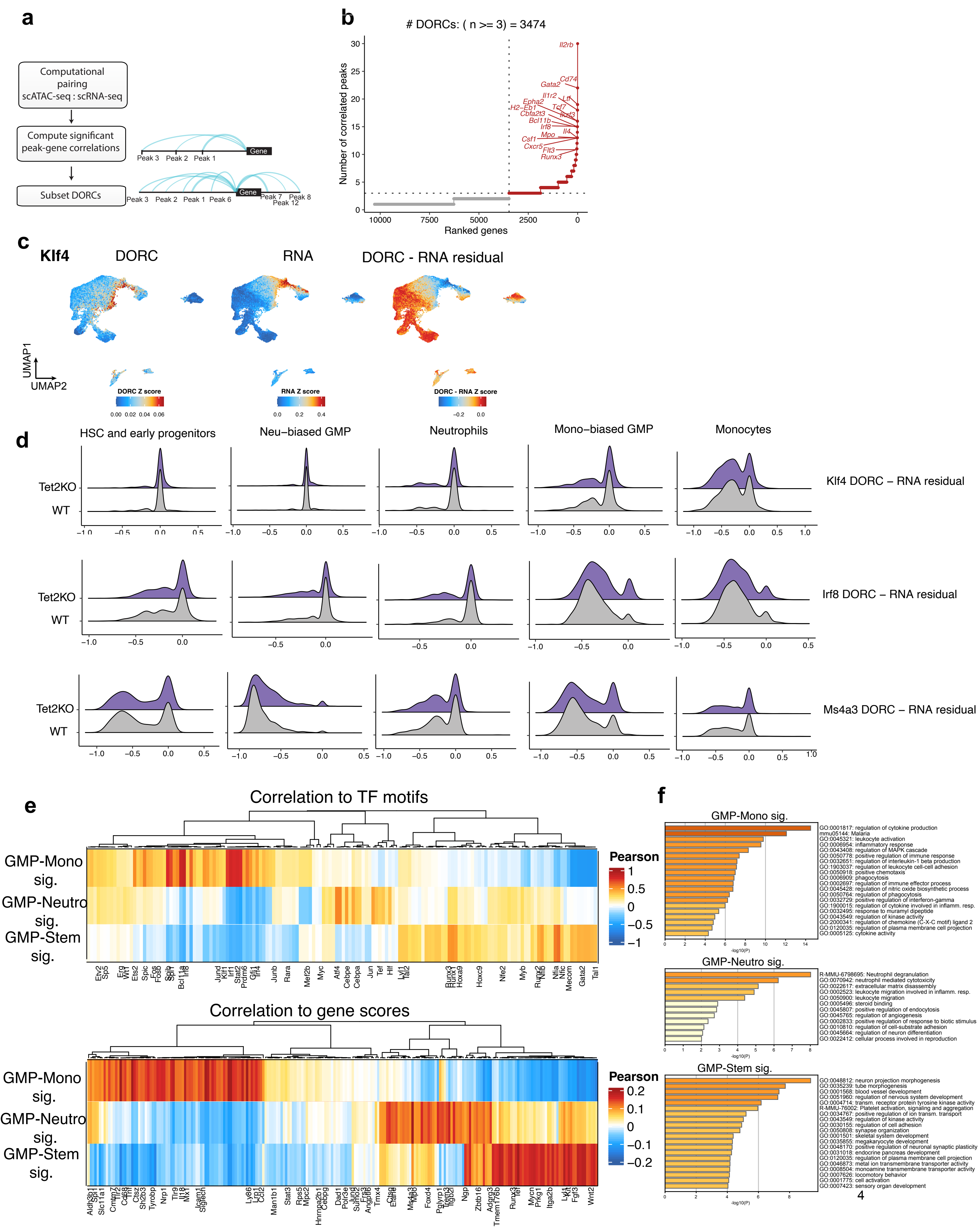
a
Computational pairing scATAC-seq : scRNA-seq
Compute significant peak-gene correlations
Subset DORCs
Gene
Peak 3 Peak 2 Peak 1
Peak 3 Peak 2 Peak 1 Peak 6 Peak 7 Peak 8 Peak 12
b
DORCs: (n >= 3) = 3474
Number of correlated peaks
Ranked genes
Il2rb
Cd74
Gata2
Il1r2
Ltf
Epha2
H2−Eb1
Tcf7
Cbfa2t3
Ikzf3
Bcl11b
Irf8
Il4
Mpo
Csf1
Cxcr5
Flt3
Runx3
c
Klf4
DORC
RNA
DORC - RNA residual
UMAP1
UMAP2
DORC Z score
RNA Z score
DORC - RNA Z score
d
HSC and early progenitors
Neu−biased GMP
Neutrophils
Mono−biased GMP
Monocytes
Tet2KO
WT
Klf4 DORC − RNA residual
Irf8 DORC − RNA residual
Ms4a3 DORC − RNA residual
e
Correlation to TF motifs
GMP-Mono sig.
GMP-Neutro sig.
GMP-Stem sig.
Pearson
Correlation to gene scores
f
GMP-Mono sig.
GO:0001817: regulation of cytokine production
mmu05144: Malaria
GO:0045321: leukocyte activation
GO:0006954: inflammatory response
GO:0043408: regulation of MAPK cascade
GO:0050778: positive regulation of immune response
GO:0032651: regulation of interleukin-1 beta production
GO:1903037: regulation of leukocyte cell-cell adhesion
GO:0050918: positive chemotaxis
GO:0006909: phagocytosis
GO:0002697: regulation of immune effector process
GO:0045428: regulation of nitric oxide biosynthetic process
GO:0050764: regulation of phagocytosis
GO:0032729: positive regulation of interferon-gamma
GO:1900015: regulation of cytokine involved in inflamm. resp.
GO:0032495: response to muramyl dipeptide
GO:0043549: regulation of kinase activity
GO:2000341: regulation of chemokine (C-X-C motif) ligand 2
GO:0120035: regulation of plasma membrane cell projection
GO:0005125: cytokine activity
GMP-Neutro sig.
R-MMU-6798695: Neutrophil degranulation
GO:0070942: neutrophil mediated cytotoxicity
GO:0022617: extracellular matrix disassembly
GO:0002523: leukocyte migration involved in inflamm. resp.
GO:0050900: leukocyte migration
GO:0005496: steroid binding
GO:0045807: positive regulation of endocytosis
GO:0045765: regulation of angiogenesis
GO:0002833: positive regulation of response to biotic stimulus
GO:0010810: regulation of cell-substrate adhesion
GO:0045664: regulation of neuron differentiation
GO:0022412: cellular process involved in reproduction
GMP-Stem sig.
GO:0048812: neuron projection morphogenesis
GO:0035239: tube morphogenesis
GO:0001568: blood vessel development
GO:0004714: transm. receptor protein tyrosine kinase activity
R-MMU-76002: Platelet activation, signaling and aggregation
GO:0034767: positive regulation of ion transm. transport
GO:0043549: regulation of kinase activity
GO:0030155: regulation of cell adhesion
GO:0050808: synapse organization
GO:0001501: skeletal system development
GO:0035855: megakaryocyte development
GO:0048170: positive regulation of neuronal synaptic plasticity
GO:0031018: endocrine pancreas development
GO:0120035: regulation of plasma membrane cell projection
GO:0046873: metal ion transmembrane transporter activity
GO:0008504: monoamine transmembrane transporter activity
GO:0007626: locomotory behavior
GO:0001775: cell activation
GO:0007423: sensory organ development
-log10(P)

Supplementary Figure 2. Chromatin signatures of GMP cells.

a) Schematics showing the computational method used for calculation of DORCs. b) Ranked identity of significant DORCs. c) UMAP plots comparing *Klf4* DORC and RNA activity. Plot on the right shows the difference between DORC accessibility and expression (DORC-RNA residual). d) DORC-RNA residual (quantile normalized, 0-99 percentile) for representative myeloid-related loci across the indicated genotypes and cell types. *Irf8* and *Ms4a3* are significantly different for Mono-biased GMP and Neutro-Biased GMP, respectively comparing *Tet2* KO and WT cells (Wilcoxon FDR < 0.0001). e) Heatmaps showing Pearson correlation of defined chromatin signatures from Fig.1e to TF motifs (top) and gene scores (bottom). f) Enrichment analysis (performed using Metascape[115]) of top correlated gene scores for each chromatin signature. Source data are provided as a Source Data file.

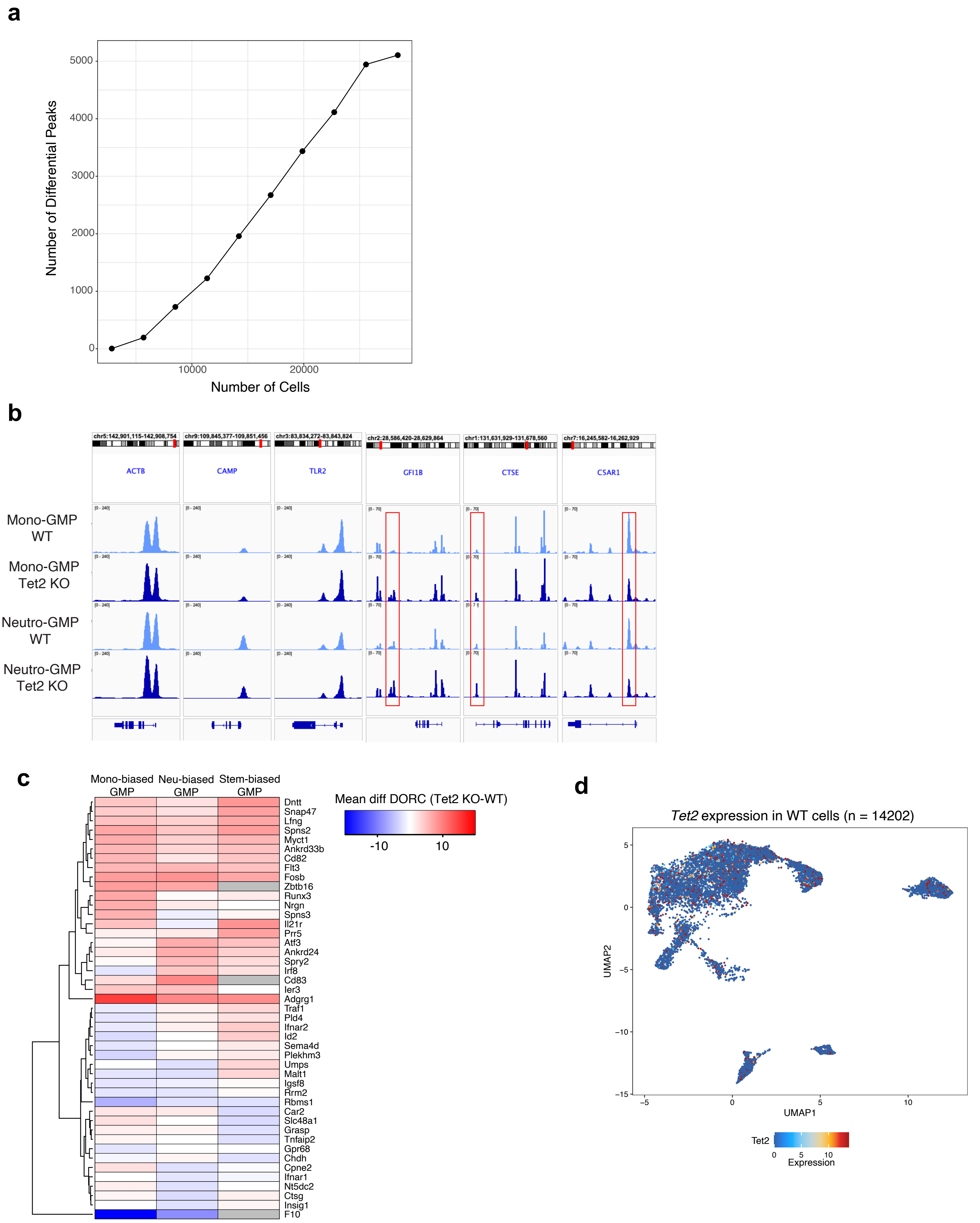
a
Number of Differential Peaks
Number of Cells
0
1000
2000
3000
4000
5000
10000
20000
b
chr5:142,901,115-142,908,754
chr9:109,845,377-109,851,456
chr3:83,834,272-83,843,824
chr2:28,586,420-28,629,864
chr1:131,631,929-131,678,560
chr7:16,245,582-16,262,929
ACTB
CAMP
TLR2
GFI1B
CTSE
C5AR1
Mono-GMP WT
Mono-GMP Tet2 KO
Neutro-GMP WT
Neutro-GMP Tet2 KO
c
Mono-biased GMP
Neu-biased GMP
Stem-biased GMP
Mean diff DORC (Tet2 KO-WT)
-10
10
Dntt
Snap47
Lfng
Spns2
Myct1
Ankrd33b
Cd82
Flt3
Fosb
Zbtb16
Runx3
Nrgn
Spns3
Il21r
Prr5
Atf3
Ankrd24
Spry2
Irf8
Cd83
Ier3
Adgrg1
Traf1
Pld4
Ifnar2
Id2
Sema4d
Plekhm3
Umps
Malt1
Igsf8
Rrm2
Rbms1
Car2
Slc48a1
Grasp
Tnfaip2
Gpr68
Chdh
Cpne2
Ifnar1
Nt5dc2
Ctsg
Insig1
F10
d
Tet2 expression in WT cells (n = 14202)
UMAP1
UMAP2
Tet2
Expression
0
5
10

Supplementary Figure 3. Differential peak and DORC analysis in *Tet2* KO cells.

a) Differential peak analysis between *Tet2* KO and WT GMPs was performed using the total amount of GMP cells available or smaller fractions to model a scenario when differential analysis was performed by individual cluster. The linear increase observed between the number of differential peaks and cells indicates that the analysis is underpowered when analyzing small cell numbers. b) Chromatin tracks showing examples of differential peaks in *Tet2* KO GMP cells (*C5ar1* and *Irf8* genes). Cells belonging to cluster 7 are included for Mono-GMP and cluster 1 for Neutro-GMP. *ActB* represents a housekeeping gene not affected by *Tet2* mutation, while *Camp* and *Tlr2* represent neutrophilic and monocytic markers, respectively. c) Heatmap showing the mean difference in single-cell DORC accessibility for top differential DORCs calculated across different GMP states, comparing *Tet2* KO and WT cells (cells belonging to cluster 7 are included for Mono-GMP, cluster 1 for Neutro-GMP, cluster 4 for Stem-GMP, utilizing only cells belonging to GMP sorted sample). Grey color indicates FDR>0.05. d) UMAP plot showing RNA expression of *Tet2* in WT mice. Source data are provided as a Source Data file.

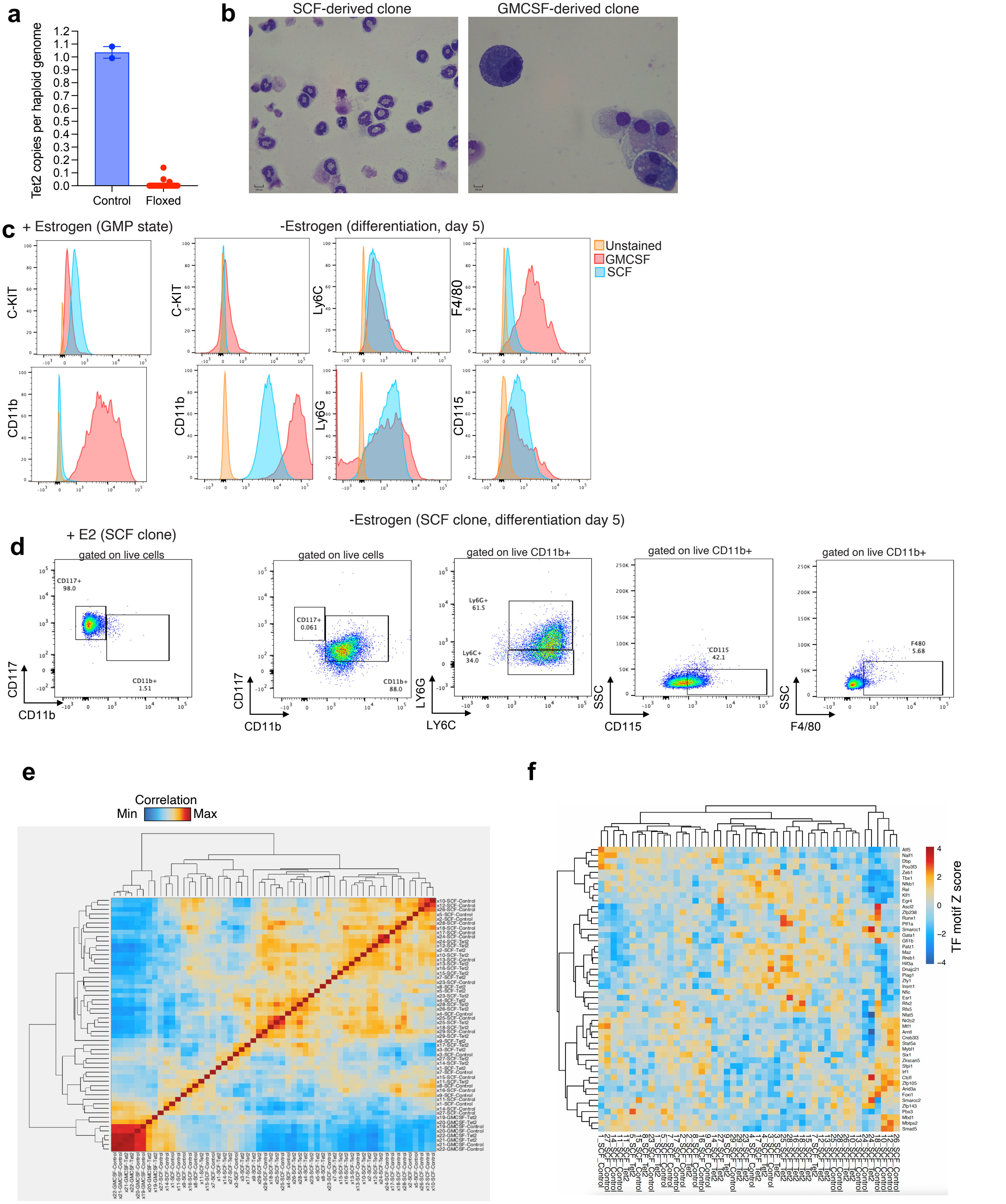
a
Tet2 copies per haploid genome
1.2
1.1
1.0
0.9
0.8
0.7
0.6
0.5
0.4
0.3
0.2
0.1
0.0
Control
Floxed
b
SCF-derived clone
GMCSF-derived clone
c
+ Estrogen (GMP state)
-Estrogen (differentiation, day 5)
Unstained
GMCSF
SCF
C-KIT
Ly6C
F4/80
CD11b
Ly6G
CD115
-Estrogen (SCF clone, differentiation day 5)
d
+ E2 (SCF clone)
gated on live cells
gated on live CD11b+
CD117+ 98.0
CD11b+ 1.51
CD117+ 0.061
CD11b+ 88.0
Ly6G+ 61.5
Ly6C+ 34.0
CD115 42.1
F480 5.68
CD117
CD11b
LY6G
LY6C
SSC
CD115
F4/80
e
Correlation
Min
Max
f
TF motif Z score

Supplementary Figure 4. Characterization of in vitro GMP clonal system.

a) qPCR showing the number of *Tet2* copies each haploid genome in WT and *Tet2* KO clones. b) Image of cytospin and Wright-Giemsa staining of representative differentiated GMP clones derived using SCF or GM-CSF cytokine as indicated at day 5 after estrogen removal. c) Representative histograms showing expression of relevant markers on GMP clones derived using SCF or GM-CSF cytokine before (+E2) or after differentiation (-E2). Expression levels of Kit indicate GMP state whether absence of Kit and expression of Cd11b indicate more differentiated cell state. d) Representative flow plots of a SCF-derived clone before differentiation (left panel) and after differentiation, showing myeloid related markers. This gating strategy has been utilized for data reported in Fig. 3c, 4j, Supplementary Fig.5. e) Pearson correlation of ATAC-seq data for all the clones analyzed in Figure 3. TF motif scores are used for this analysis. Clone number, genotype (control, *Tet2*) and cell type (SCF, GM-CSF) are indicated. f) Heatmap reporting top TF motifs associated with variability in chromatin accessibility between individual SCF-derived clones. This analysis highlights which specific features contribute to the heterogeneity observed within SCF clones from Supplementary Fig.4e. Source data are provided as a Source Data file.

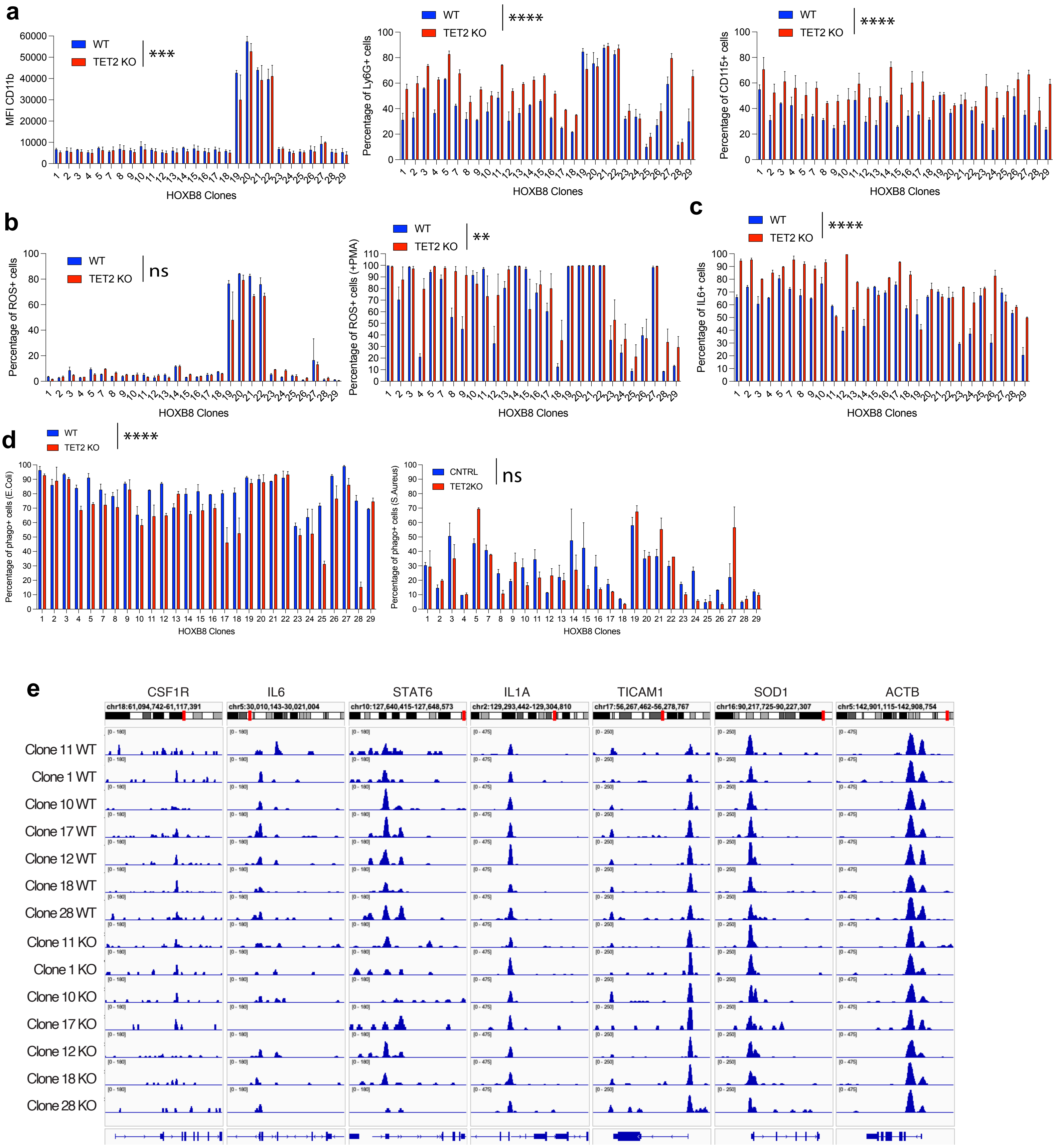
a
MFI CD11b
WT
TET2 KO
***
HOXB8 Clones
Percentage of Ly6G+ cells
****
Percentage of CD115+ cells
****
b
Percentage of ROS+ cells
ns
Percentage of ROS+ cells (+PMA)
**
c
Percentage of IL6+ cells
****
d
Percentage of phago+ cells (E.Coli)
****
Percentage of phago+ cells (S.Aureus)
CNTRL
TET2KO
ns
e
CSF1R
IL6
STAT6
IL1A
TICAM1
SOD1
ACTB
chr18:61,094,742-61,117,391
chr5:30,010,143-30,021,004
chr10:127,640,415-127,648,573
chr2:129,293,442-129,304,810
chr17:56,267,462-56,278,767
chr16:90,217,725-90,227,307
chr5:142,901,115-142,908,754
Clone 11 WT
Clone 1 WT
Clone 10 WT
Clone 17 WT
Clone 12 WT
Clone 18 WT
Clone 28 WT
Clone 11 KO
Clone 1 KO
Clone 10 KO
Clone 17 KO
Clone 12 KO
Clone 18 KO
Clone 28 KO

Supplementary Figure 5. Functional and molecular assays define heterogeneity within WT and *Tet2* KO GMP clones.

Functional assays performed on single GMP clone pairs 5 days after differentiation into mature effectors. Genotype is color coded. Statistics are calculated using two-tailed Wilcoxon matched-pairs signed rank test. a) Histograms showing for each matched WT and *Tet2* KO GMP clone pair levels of Cd11b (left, p=0.001), Ly6G (middle, p<0.0001) and Cd115 (right, p<0.0001) n=3 technical replicates for each clone pair. b) Histograms showing for each matched WT and *Tet2* KO GMP clone pair levels of ROS expression in a basal state (left, p=0.493) and after stimulation with PMA (right, p=0.0089) n=2 technical replicates for each clone pair. c) Histograms showing for each matched WT and *Tet2* KO GMP clone pair IL6 production after stimulation with LPS (p<0.0001). n=3 technical replicates for each clone pair. d) Histograms showing for each matched WT and *Tet2* KO GMP clone pair levels of phagocytosis when cells were challenged with E.Coli bioparticles (left, p<0.0001) or with S.Aureus bioparticles (right, p=0.338) n=2 technical replicates for each clone pair. e) Chromatin tracks showing examples of differential peaks in selected SCF WT clones. High and low performers in functional assays from Figure 3C are selected. *Csf1r* (gene coding for Cd115) and *Il6* are differential loci emerged from functional assays. *Stat6*, *Il1a* and *Ticam1* are representative genes involved in inflammatory response of myeloid cells. *Sod1* is a negative regulator of ROS production. *ActB* represents a housekeeping gene. Corresponding *Tet2* KO clones are shown on the bottom. Source data are provided as a Source Data file.

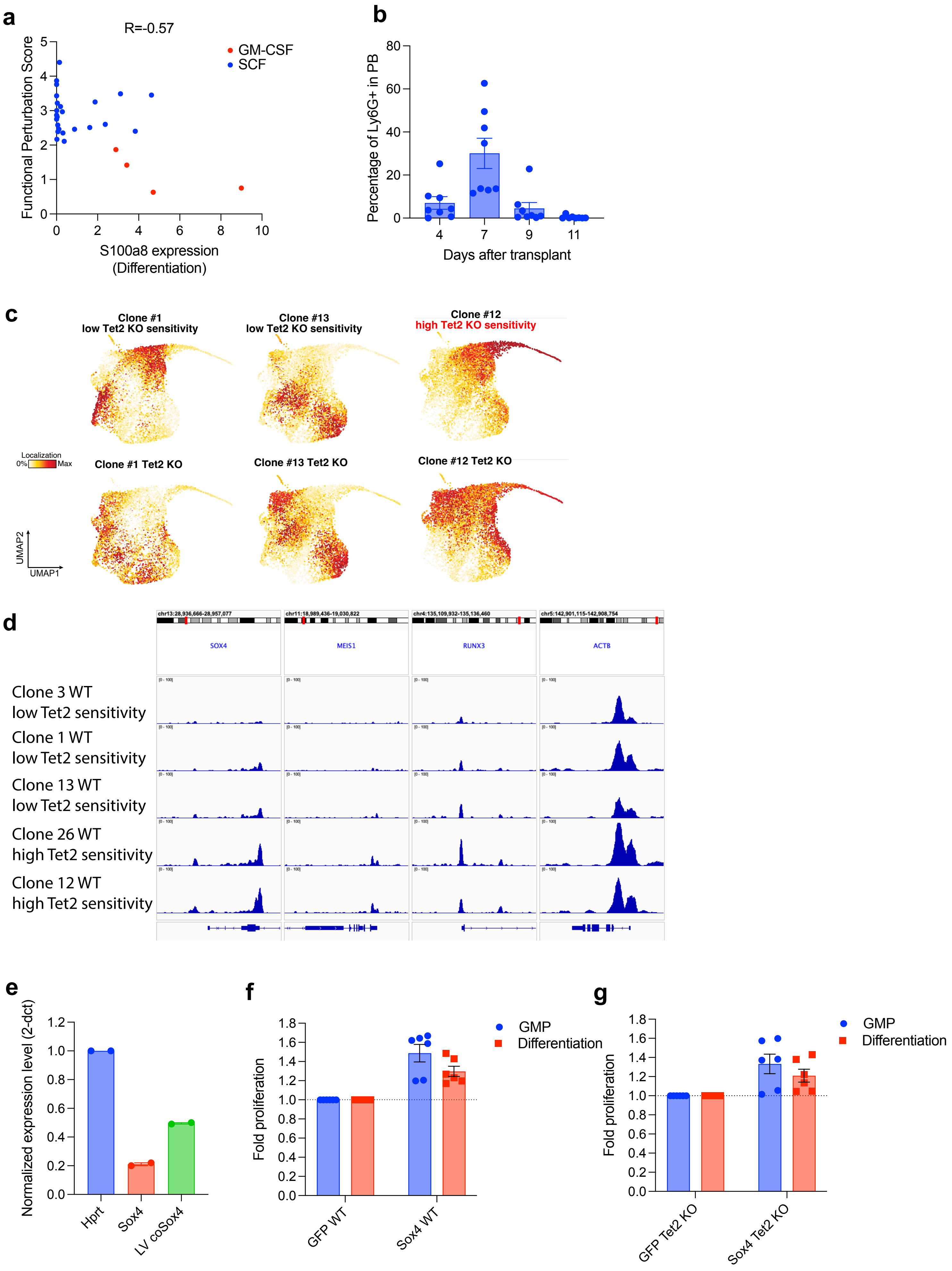

a
R=-0.57
GM-CSF
SCF
Functional Perturbation Score
S100a8 expression
(Differentiation)
b
Percentage of Ly6G+ in PB
Days after transplant
c
Clone #1
low Tet2 KO sensitivity
Clone #13
low Tet2 KO sensitivity
Clone #12
high Tet2 KO sensitivity
Localization
0%
Max
Clone #1 Tet2 KO
Clone #13 Tet2 KO
Clone #12 Tet2 KO
UMAP2
UMAP1
d
chr13:28,936,666-28,957,077
chr11:18,989,436-19,030,822
chr4:135,109,932-135,136,460
chr5:142,901,115-142,908,754
SOX4
MEIS1
RUNX3
ACTB
Clone 3 WT
low Tet2 sensitivity
Clone 1 WT
low Tet2 sensitivity
Clone 13 WT
low Tet2 sensitivity
Clone 26 WT
high Tet2 sensitivity
Clone 12 WT
high Tet2 sensitivity
e
Normalized expression level (2-dct)
Hprt
Sox4
LV coSox4
f
Fold proliferation
GMP
Differentiation
GFP WT
Sox4 WT
g
Fold proliferation
GMP
Differentiation
GFP Tet2 KO
Sox4 Tet2 KO

Supplementary Figure 6. Chromatin heterogeneity within SCF clones defines sensitization status to *Tet2* KO.

a) Pearson Correlation coefficient comparing the Functional Perturbation score and transcript levels of *S100a8* in undifferentiated GMP clones. n=28. *S100a8* represents a marker of myeloid differentiation[54]. GM-CSF and SCF clones are color-coded. b) Percent output in the PB at different time points after transplant of SCF GMP clones in lethally irradiated recipient mice. n=8. c) UMAP plots showing single cell distribution of WT and mutated GMP clone pairs analyzed by SHARE seq. d) Representative ATAC seq tracks showing regulatory region of *Sox4* and *Actb* genes in different GMP clones endowed with different levels of functional alterations observed after Tet2 mutation e) Quantitative PCR showing expression of *Sox4* from endogenous transcript (Sox4) or from the overexpressing LV (LV- codon optimized Sox4). Data are normalized to the expression of *Hprt*. f) Fold change in the cell number comparing control (GFP overexpressing) and *Sox4* overexpressing WT clones.n=6. Measurements are performed 5 days after plating equal cell number under GMP maintaining condition or differentiating conditions. g) Same as in F, comparing *Tet2* KO clones. n=6. Source data are provided as a Source Data file.

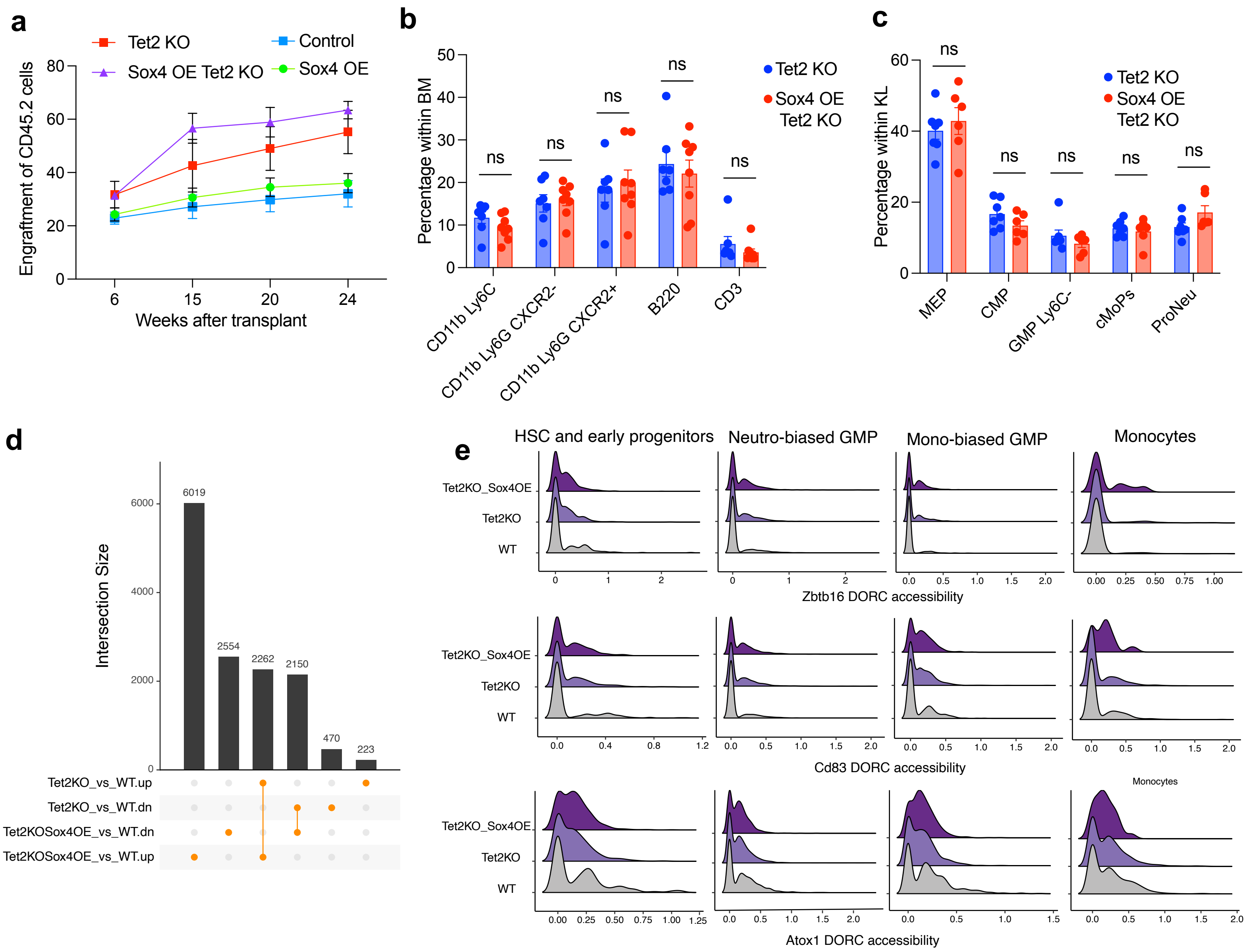
a
Tet2 KO
Control
Sox4 OE Tet2 KO
Sox4 OE
Engraftment of CD45.2 cells
Weeks after transplant
b
Percentage within BM
Tet2 KO
Sox4 OE Tet2 KO
ns
CD11b Ly6C
CD11b Ly6G CXCR2-
CD11b Ly6G CXCR2+
B220
CD3
c
Percentage within KL
Tet2 KO
Sox4 OE Tet2 KO
ns
MEP
CMP
GMP Ly6C-
cMoPs
ProNeu
d
Intersection Size
6019
2554
2262
2150
470
223
Tet2KO_vs_WT.up
Tet2KO_vs_WT.dn
Tet2KOSox4OE_vs_WT.dn
Tet2KOSox4OE_vs_WT.up
e
HSC and early progenitors
Neutro-biased GMP
Mono-biased GMP
Monocytes
Tet2KO_Sox4OE
Tet2KO
WT
Zbtb16 DORC accessibility
Cd83 DORC accessibility
Atox1 DORC accessibility

Supplementary Figure 7. Phenotypic and molecular characterization of *Sox4* OE *Tet2* KO cells.

a) Percentage of CD45.2 cells measured at different time points in the PB of mice transplanted with GFP overexpression vector in presence (*Tet2* KO) or absence (control) of inducible *Tet2* KO cells as compared to cells with Sox4 overexpression vector in presence (*Sox4* OE *Tet2* KO) or absence (*Sox4* OE) of inducible *Tet2* KO (n=3). To induce *Tet2* deletion, pI:pC was administered 6 weeks after transplant. B) Distribution of the indicated differentiated populations in the BM of mice from Fig.5B 35 weeks after transplant (n=7 *Tet2* KO, n=8 *Sox4* OE *Tet2* KO). Mann-Whitney test; p>0.05. c) Distribution of the indicated primitive progenitor populations within Lin-Kit+Sca- cells in the BM of mice from Fig.5B 35 weeks after transplant (n=7 *Tet2* KO, n=8 *Sox4* OE *Tet2* KO). Mann-Whitney test; p>0.05. Populations are defined as in[44] d) Barplot showing the overlap between differential peaks from scATACseq dataset comparing *Tet2* KO and *Sox4* OE *Tet2* KO cells as respect to WT. e) DORC accessibility (quantile normalized, 0-99 percentile) for representative myeloid-related loci across the indicated genotypes and cell types. All loci are significantly altered in *Tet2* KO (Wilcoxon FDR < 0.0001, see Supplementary Data 3). Increased accessibility at *Zbtb16* locus was previously reported in *Tet2* KO primitive cells[45]. Source data are provided as a Source Data file.

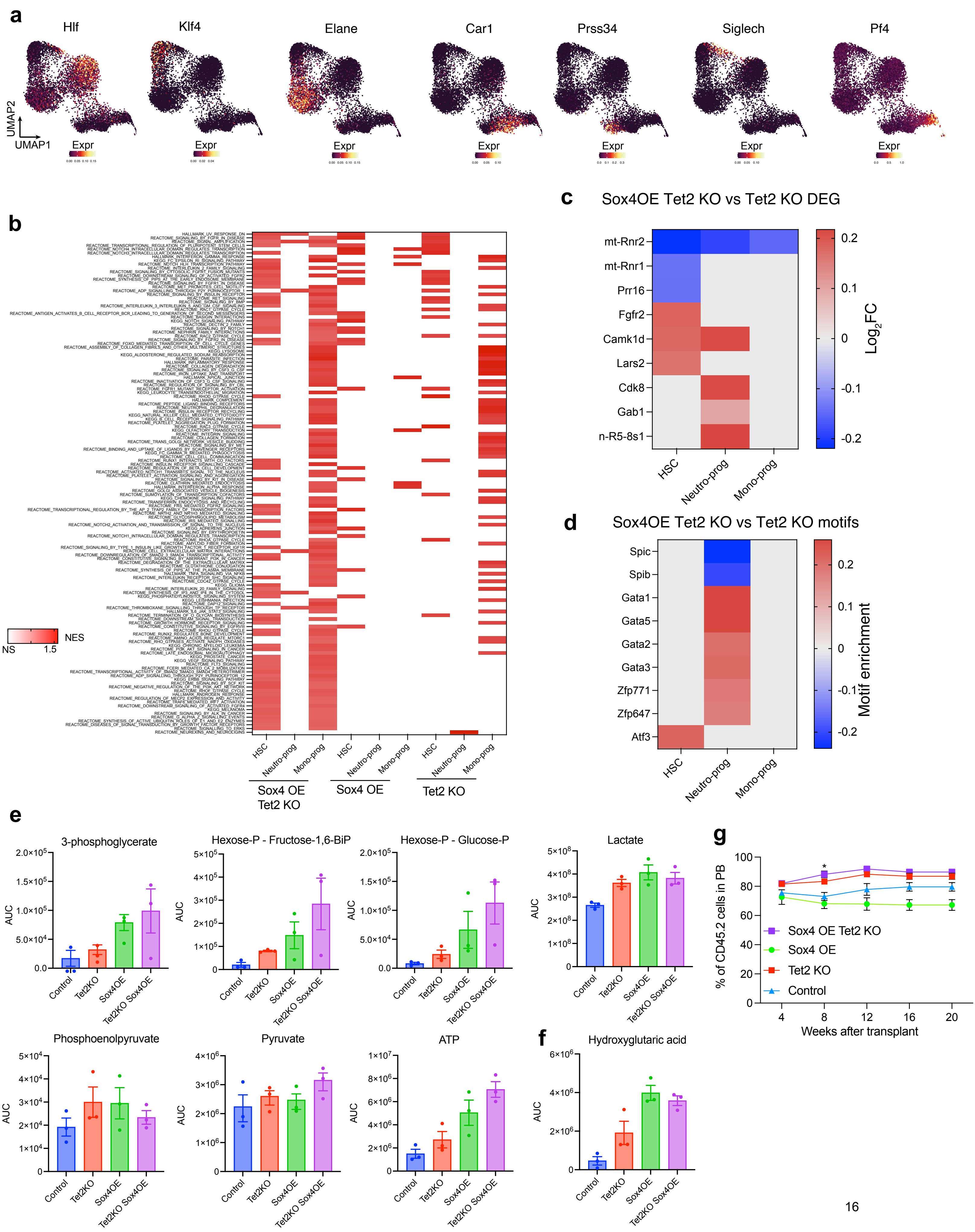
a
Hlf
Klf4
Elane
Car1
Prss34
Siglech
Pf4
UMAP2
UMAP1
Expr
b
NES
NS
1.5
HSC
Neutro-prog
Mono-prog
Sox4 OE Tet2 KO
Sox4 OE
Tet2 KO
c
Sox4OE Tet2 KO vs Tet2 KO DEG
mt-Rnr2
mt-Rnr1
Prr16
Fgfr2
Camk1d
Lars2
Cdk8
Gab1
n-R5-8s1
Log2FC
d
Sox4OE Tet2 KO vs Tet2 KO motifs
Spic
Spib
Gata1
Gata5
Gata2
Gata3
Zfp771
Zfp647
Atf3
Motif enrichment
e
3-phosphoglycerate
Hexose-P - Fructose-1,6-BiP
Hexose-P - Glucose-P
Lactate
Phosphoenolpyruvate
Pyruvate
ATP
AUC
Control
Tet2KO
Sox4OE
Tet2KO Sox4OE
f
Hydroxyglutaric acid
g
% of CD45.2 cells in PB
Weeks after transplant
Sox4 OE Tet2 KO
Sox4 OE
Tet2 KO
Control

Supplementary Figure 8. Functional effects of *Sox4* OE *Tet2* KO in an HSC-based model.

a) UMAP plots showing RNA expression of representative markers utilized for annotating PVA-expanded cells from Fig.6c. b) GSEA analysis comparing gene expression data from Fig.6c. Each sample is compared to WT control cells. HSC cluster, Neutro and Mono progenitor clusters are shown. c) Differential genes from Fig.6c (Pval adj<0.05) comparing *Sox4* OE *Tet2* KO and *Tet2* KO among the indicated clusters. d) TF motif analysis showing *Sox4* OE *Tet2* KO vs *Tet2* KO comparison within the indicated cell clusters from Fig.6c. TF motifs with FDR<0.25 are shown. e) GMP clones (n=3) treated as indicated were analyzed by untargeted metabolomics. Normalized AUC (Area Under Curve) of different metabolites related to the glycolysis pathway are shown. f) Normalized AUC of hydroxyglutaric acid (2-HG) in GMP clones from Supplementary Fig.8e. g) Percentage of CD45.2 cells measured at different time points in the PB after primary BM transplantation of cells from Fig.6h. n=3. Two-way Anova with Dunnett's multiple comparison test, p<0.05. Source data are provided as a Source Data file.

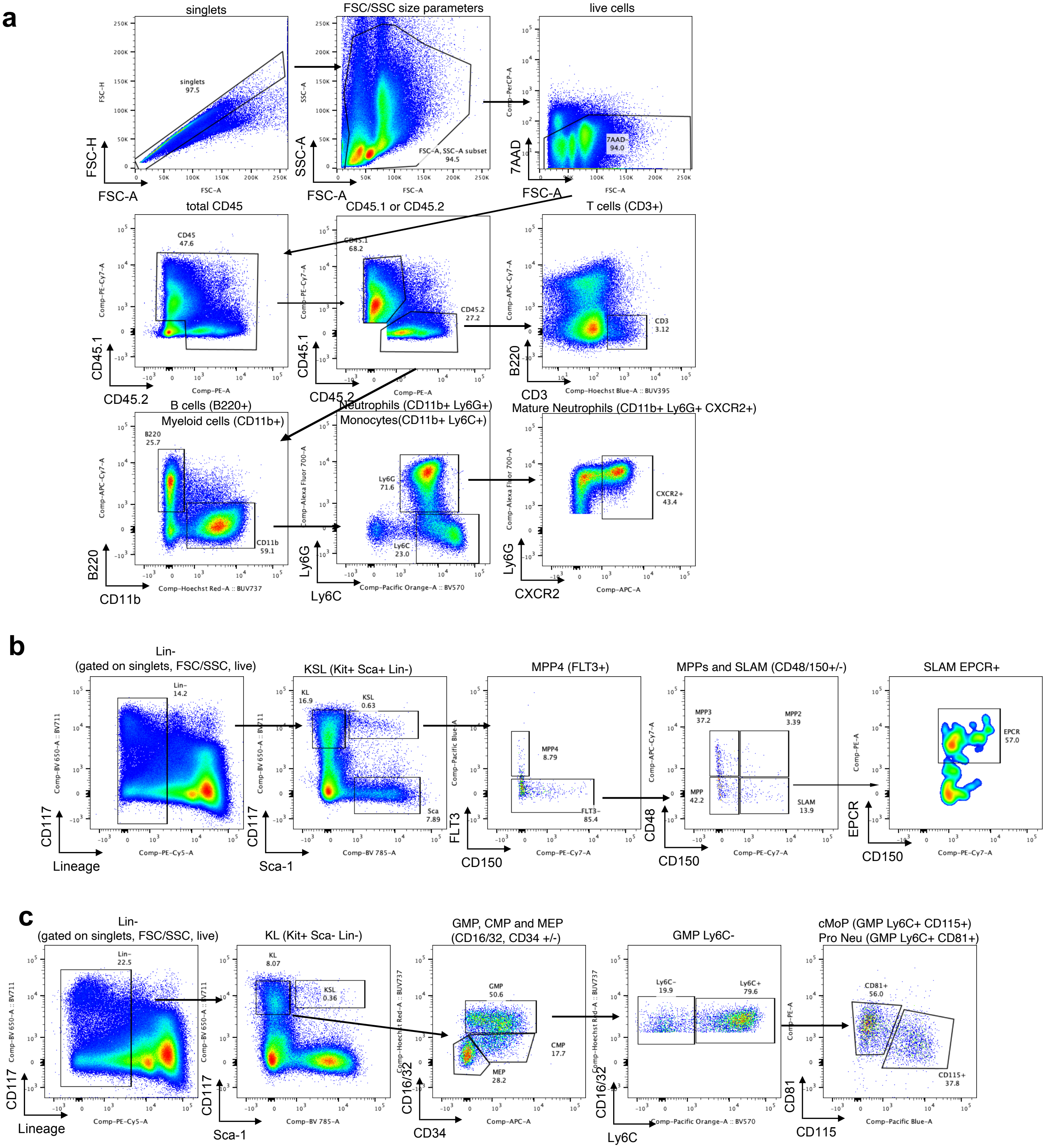
a
singlets
FSC/SSC size parameters
live cells
singlets 97.5
FSC-A, SSC-A subset 94.5
7AAD- 94.0
FSC-H
FSC-A
SSC-A
7AAD
total CD45
CD45.1 or CD45.2
T cells (CD3+)
CD45 47.6
CD45.1 68.2
CD45.2 27.2
CD3 3.12
CD45.1
CD45.2
B220
CD3
B cells (B220+)
Myeloid cells (CD11b+)
Neutrophils (CD11b+ Ly6G+)
Monocytes(CD11b+ Ly6C+)
Mature Neutrophils (CD11b+ Ly6G+ CXCR2+)
B220 25.7
CD11b 59.1
Ly6G 71.6
Ly6C 23.0
CXCR2+ 43.4
CD11b
Ly6G
Ly6C
CXCR2
b
Lin-
(gated on singlets, FSC/SSC, live)
KSL (Kit+ Sca+ Lin-)
MPP4 (FLT3+)
MPPs and SLAM (CD48/150+/-)
SLAM EPCR+
Lin- 14.2
KL 16.9
KSL 0.63
Sca 7.89
MPP4 8.79
FLT3- 85.4
MPP3 37.2
MPP2 3.39
MPP 42.2
SLAM 13.9
EPCR 57.0
CD117
Lineage
Sca-1
FLT3
CD150
CD48
EPCR
c
Lin-
(gated on singlets, FSC/SSC, live)
KL (Kit+ Sca- Lin-)
GMP, CMP and MEP
(CD16/32, CD34 +/-)
GMP Ly6C-
cMoP (GMP Ly6C+ CD115+)
Pro Neu (GMP Ly6C+ CD81+)
Lin- 22.5
KL 8.07
KSL 0.36
GMP 50.6
CMP 17.7
MEP 28.2
Ly6C- 19.9
Ly6C+ 79.6
CD81+ 56.0
CD115+ 37.8
CD117
Lineage
Sca-1
CD16/32
CD34
Ly6C
CD81
CD115

Supplementary Figure 9. Gating strategies for flow cytometry.

a) Representative gating strategy used to define PB and BM differentiated mature cells. Used in Fig.4k, 5b, 6h, Supplementary Fig. 1b, 6b, 7a, 7b, 8g. b) Representative gating strategy used to define HSC and early progenitor populations. When used in primary mouse BM, additional CD45.1/CD45.2 pre-gating as reported in panel a was added. Used in Fig. 5c, 6b, Supplementary Fig. 1e. c) Representative gating strategy used to define early myeloid progenitor populations. When used in primary mouse BM, additional CD45.1/CD45.2 pre-gating as reported in panel a was added. Used in Supplementary Fig. 7c.
