# Supplementary material for: Cell of origin epigenetic priming determines susceptibility to Tet2 mutation
Source: Nat Commun. 2024 May 21;15:4325. doi: 10.1038/s41467-024-48508-6 (PMC11109152; doi:10.1038/s41467-024-48508-6)
Supplement: Supplementary file 3 — Description of Additional Supplementary Files [file 41467_2024_48508_MOESM3_ESM.pdf]

Description of Supplementary Data

Cell of origin epigenetic priming determines susceptibility to Tet2 mutation.

Schiroli et al.

Supplementary Data 1. scATAC and RNA dataset QC numbers for each individual sample and mouse.

Supplementary Data 2. Epigenetic GMP signatures.
Page 1: Correlation between GMP modules and DORCs.
Page 2: Correlation between GMP modules and Gene scores.
Page 3: Correlation between GMP modules and TF motifs.
Page 4: Peak coordinates (mm10) associated to GMP modules.

Supplementary Data 3. Differential DORCs in GMP populations.
Page 1: Differential DORCs comparing Tet2 KO and WT Neutro-biased GMP
Page 2: Differential DORCs comparing Tet2 KO and WT Mono-biased GMP
Page 3: Differential DORCs comparing Tet2 KO and WT Stem-biased GMP

Supplementary Data 4. List of DNA oligonucleotides utilized and description.

Supplementary Data 5. List of Antibodies utilized and description.

Supplementary Data 6. Normalized AUC and relative detected compounds from untargeted metabolomics analysis.
