# Supplementary material for: Cell of origin epigenetic priming determines susceptibility to Tet2 mutation
Source: Nat Commun. 2024 May 21;15:4325. doi: 10.1038/s41467-024-48508-6 (PMC11109152; doi:10.1038/s41467-024-48508-6)
Supplement: Supplementary file 10 — Reporting Summary [file 41467_2024_48508_MOESM10_ESM.pdf]

Corresponding author(s): David Scadden, MD

Last updated by author(s): 04/12/2024

Reporting Summary

Nature Portfolio wishes to improve the reproducibility of the work that we publish. This form provides structure for consistency and transparency in reporting. For further information on Nature Portfolio policies, see our Editorial Policies and the Editorial Policy Checklist.

Statistics

For all statistical analyses, confirm that the following items are present in the figure legend, table legend, main text, or Methods section.

n/a	Confirmed	
☐	☒	The exact sample size (*n*) for each experimental group/condition, given as a discrete number and unit of measurement
☐	☒	A statement on whether measurements were taken from distinct samples or whether the same sample was measured repeatedly
☐	☒	The statistical test(s) used AND whether they are one- or two-sided<br>*Only common tests should be described solely by name; describe more complex techniques in the Methods section.*
☐	☒	A description of all covariates tested
☐	☒	A description of any assumptions or corrections, such as tests of normality and adjustment for multiple comparisons
☐	☒	A full description of the statistical parameters including central tendency (e.g. means) or other basic estimates (e.g. regression coefficient) AND variation (e.g. standard deviation) or associated estimates of uncertainty (e.g. confidence intervals)
☐	☒	For null hypothesis testing, the test statistic (e.g. *F*, *t*, *r*) with confidence intervals, effect sizes, degrees of freedom and *P* value noted<br>*Give P values as exact values whenever suitable.*
☒	☐	For Bayesian analysis, information on the choice of priors and Markov chain Monte Carlo settings
☒	☐	For hierarchical and complex designs, identification of the appropriate level for tests and full reporting of outcomes
☐	☒	Estimates of effect sizes (e.g. Cohen's *d*, Pearson's *r*), indicating how they were calculated

Our web collection on statistics for biologists contains articles on many of the points above.

Software and code

Policy information about availability of computer code

Data collection	Described in the manuscript.
Data analysis	sc-ATACseq and sc-RNAseq dataset can be explored using a custom interactive application (https://buenrostrolab.shinyapps.io/gmps/).<br>The code utilized for data analysis is available at https://github.com/buenrostrolab/GMP_analyses_code<br>Statistical analysis was performed by using Graphpad Prism (Version 9.0.0). Bioinformatic analysis was performed by using R language (Version 4.3.2). Genome-wide chromatin accessibility peaks were called using MACS v2, For ATAC-related analyses, chromVAR R package (v1.12.0) and FigR R package (v1.0.1) were used. For sc-RNAseq data processing, Cellranger v3.1.0 was used. For scRNA-seq data analysis, Seurat R package v4 was used.

For manuscripts utilizing custom algorithms or software that are central to the research but not yet described in published literature, software must be made available to editors and reviewers. We strongly encourage code deposition in a community repository (e.g. GitHub). See the Nature Portfolio guidelines for submitting code & software for further information.

Data

Policy information about availability of data

All manuscripts must include a data availability statement. This statement should provide the following information, where applicable:

- Accession codes, unique identifiers, or web links for publicly available datasets
- A description of any restrictions on data availability
- For clinical datasets or third party data, please ensure that the statement adheres to our policy

scRNAseq, scATACseq and bulk ATACseq datasets are deposited in the GEO database under accession GSE247970 [https://www.ncbi.nlm.nih.gov/geo/query/acc.cgi?acc=GSE247970]. sc-ATACseq and sc-RNAseq processed datasets can be also explored using a custom interactive application [https://buenrostrolab.shinyapps.io/gmps/]. Metabolomics sata are deposited in the MetaboLights database under accession MTBLS9914 [https://www.ebi.ac.uk/metabolights/MTBLS9914].

Research involving human participants, their data, or biological material

Policy information about studies with human participants or human data. See also policy information about sex, gender (identity/presentation), and sexual orientation and race, ethnicity and racism.

Reporting on sex and gender	N/A
Reporting on race, ethnicity, or other socially relevant groupings	N/A
Population characteristics	N/A
Recruitment	N/A
Ethics oversight	N/A

Note that full information on the approval of the study protocol must also be provided in the manuscript.

Field-specific reporting

Please select the one below that is the best fit for your research. If you are not sure, read the appropriate sections before making your selection.

☒ Life sciences ☐ Behavioural & social sciences ☐ Ecological, evolutionary & environmental sciences

For a reference copy of the document with all sections, see nature.com/documents/nr-reporting-summary-flat.pdf

Life sciences study design

All studies must disclose on these points even when the disclosure is negative.

Sample size	No sample size calculation was performed. For in vivo and in vitro studies, sample sizes were determined based on our previous publications and preliminary experiments (at least three lines were used each in vitro and at least 3 independent mice were used each in vivo experiment). Sample sizes were chosen based on empirical values that were sufficient to detect meaningful biological differences with good reproducibility. Sample size is indicated in the legends or text.
Data exclusions	No data were excluded from analysis.
Replication	Sequencing was replicated on at least 2 mice each group, and on n>3 immortalized lines, depending on sample availability. All attempts at replication gave similar results.
Randomization	Randomization was not performed due to inherent differences in mouse genotypes. Age- and sex-matched mice of different genotypes were used for animal experiments as reported in the manuscript. All mice that were used as recipients in bone marrow transplantation experiments were female, 8-12 weeks of age.
Blinding	Not relevant for objective measures.

Reporting for specific materials, systems and methods

We require information from authors about some types of materials, experimental systems and methods used in many studies. Here, indicate whether each material, system or method listed is relevant to your study. If you are not sure if a list item applies to your research, read the appropriate section before selecting a response.

Materials & experimental systems

n/a	Involved in the study
☐	☒ Antibodies
☐	☒ Eukaryotic cell lines
☒	☐ Palaeontology and archaeology
☐	☒ Animals and other organisms
☒	☐ Clinical data
☒	☐ Dual use research of concern
☒	☐ Plants

Methods

n/a	Involved in the study
☒	☐ ChIP-seq
☐	☒ Flow cytometry
☒	☐ MRI-based neuroimaging

Antibodies

Antibodies used

List of antibodies used is provided in Supplementary Data 5 file.
For each line, Antigen with relative species and fluorochrome, Vendor with catalog number, Dilution are reported.
Antigen and fluorochrome Vendor Identifier (catalog and RRID) Dilution
Anti mouse CD45.2 PE Biolegend Cat# 109808 1/200
Anti mouse CD45.2 BUV395 BD Biosciences Cat# 564616 1/200
Anti mouse CD45.2 APC Biolegend Cat# 109814 1/200
Anti mouse CD45.1 APC-Cy7 BD Biosciences Cat# 560579 1/200
Anti mouse CD45.1 BUV3995 BD Biosciences Cat# 565212 1/200
Anti-mouse CD19 BV786 BD Biosciences Cat# 563333 1/200
Anti-mouse Ly6C BV570 Biolegend Cat# 128030 1/200
Anti-mouse Ly6C BV421 Biolegend Cat# 128032 1/200
Anti-mouse Ly6G AF700 BD Biosciences Cat# 561236 1/200
Anti-mouse Ly-6A/E BV785 Biolegend Cat# 108139 1/200
Anti-mouse CD16/32 BVU737 BD Biosciences Cat# 612783 1/200
Anti-mouse Ly6G/Ly6C Biotin BD Biosciences Cat# 553124 1/200
Anti-mouse CD117 BV421 Biolegend Cat# 135123 1/100
Anti-mouse CD117 BV711 Biolegend Cat# 105835 1/100
Anti-mouse CD117 BUV395 BD Biosciences Cat# 564011 1/100
Anti-mouse CD3e BUV395 BD Biosciences Cat# 563565 1/200
Anti-mouse CD3e Biotin BD Biosciences Cat# 553060 1/200
Anti-mouse CD4 Biotin BD Biosciences Cat# 553728 1/200
Anti-mouse CD8 Biotin BD Biosciences Cat# 553029 1/200
Anti-mouse CD115 BV421 Biolegend Cat# 135513 1/100
Anti-mouse CD11b Pecy7 Biolegend Cat# 101216 1/100
Anti-mouse CD11b BUV805 BD Biosciences Cat# 742004 1/100
Anti-mouse CD11b Biotin BD Biosciences Cat# 553309 1/100
Anti-mouse CD45R/B220 BV785 Biolegend Cat# 103246 1/200
Anti-mouse CD45R/B220 Biotin BD Biosciences Cat# 553086 1/200
Anti-mouse Ter119 Biotin BD Biosciences Cat# 553672 1/200
Anti-mouse CD48 PB Biolegend Cat# 103418 1/200
Anti-mouse CD150 PE Biolegend Cat# 115904 1/100
Anti-mouse CD150 Pecy7 Biolegend Cat# 115914 1/100
Anti-mouse CD34 APC Biolegend Cat# 119310 1/20 ul
Anti-mouse CD71 PE Biolegend Cat# 113808 1/100
Anti-mouse F4/80 APC-Cy7 Biolegend Cat# 123117 1/50 ul
Streptavidin BUV395 BD Biosciences Cat# 564176 1/100
Streptavidin BV711 BD Biosciences Cat# 563262 1/100
Anti-mouse IL6 PE Biolegend Cat# 504504 1/100
Anti-mouse CD182 APC Biolegend Cat# 149311 1/100
Anti-mouse CD135 BV421 BD Biosciences Cat# 562898 1/100
Anti-mouse CD201 PE Biolegend Cat# 141504 1/50 ul

Validation

All antibodies used are commercially available and validated by the manufacturers.
For each line, Antibody and relative RRID validation numbers are reported
Anti mouse CD45.2 PE RRID:AB_313445
Anti mouse CD45.2 BUV395 RRID:AB_2738867
Anti mouse CD45.2 APC RRID:AB_389211
Anti mouse CD45.1 APC-Cy7 RRID:AB_1727487
Anti mouse CD45.1 BUV3995 RRID:AB_2722493
Anti-mouse CD19 BV786 RRID:AB_2738141
Anti-mouse Ly6C BV570 RRID:AB_2562617
Anti-mouse Ly6C BV421 RRID:AB_2562178
Anti-mouse Ly6G AF700 RRID:AB_10611860
Anti-mouse Ly-6A/E BV785 RRID:AB_2565957
Anti-mouse CD16/32 BVU737 RRID:AB_2870112
Anti-mouse Ly6G/Ly6C Biotin RRID:AB_394640
Anti-mouse CD117 BV421 RRID:AB_2562236
Anti-mouse CD117 BV711 RRID:AB_2565956

Anti-mouse CD117 BUV395 RRID:AB_2738541
Anti-mouse CD3e BUV395 RRID:AB_2738278
Anti-mouse CD3e Biotin RRID:AB_394593
Anti-mouse CD4 Biotin RRID:AB_395012
Anti-mouse CD8 Biotin RRID:AB_394567
Anti-mouse CD115 BV421 RRID:AB_2562667
Anti-mouse CD11b Pecy7 RRID:AB_312799
Anti-mouse CD11b BUV805 RRID:AB_2871303
Anti-mouse CD11b Biotin RRID:AB_394773
Anti-mouse CD45R/B220 RRID:AB_2563256
Anti-mouse CD45R/B220 Biotin RRID:AB_394616
Anti-mouse Ter119 Biotin RRID:AB_394985
Anti-mouse CD48 PB RRID:AB_756140
Anti-mouse CD150 PE RRID:AB_313683
Anti-mouse CD150 Pecy7 RRID:AB_439797
Anti-mouse CD34 APC RRID:AB_1236469
Anti-mouse CD71 PE RRID:AB_313569
Anti-mouse F4/80 APC-Cy7 RRID:AB_893489
Streptavidin BUV395 RRID:AB_2869553
Streptavidin BV711 RRID:AB_2869478
Anti-mouse IL6 PE RRID:AB_315338
Anti-mouse CD182 APC RRID:AB_2728184
Anti-mouse CD135 BV421 RRID:AB_2737876
Anti-mouse CD201 RRID:AB_10895909

Eukaryotic cell lines

Policy information about cell lines and Sex and Gender in Research

Cell line source(s)	Hoxb8-ER immortalized lines were derived from Tet2 fl/fl mice (The Jackson Laboratory, #017573). Lines from both male and female mice were used in this study.
Authentication	Identity of lines was confirmed by flow cytometry (expression of GMP markers), genotype was confirmed by ddPCR.
Mycoplasma contamination	All cell line used in this study tested negative for Mycoplasma.
Commonly misidentified lines (See ICLAC register)	None used

Animals and other research organisms

Policy information about studies involving animals; ARRIVE guidelines recommended for reporting animal research, and Sex and Gender in Research

Laboratory animals	Tet2 fl/fl mice were obtained from The Jackson Laboratory (#017573) and crossed to Mx1-Cre mice (#003556, The Jackson Laboratory). Mx1-Cre negative littermates were utilized as controls. For bone marrow transplantation experiments, aged-matched 8-12 weeks old CD45.1(STEM) mice and WT C57BL6/J recipients (#000664, The Jackson Laboratory) were utilized.<br>Animals were housed at 70 °F-74 °F in the facilities from Massachusetts General Hospital. The light cycle of animal rooms is 12 hours of light and 12 hours of dark. All mice were bred and maintained in pathogen-free conditions and all procedures performed were approved by the Institutional Animal Care and Use Committee of Massachusetts General Hospital (protocol #2016N000085).
Wild animals	The study did not involve wild animals.
Reporting on sex	8-12 weeks old, age-matched randomized male and female mice were used in all experiments. For bone marrow transplantation experiments, female mice were used as recipients.
Field-collected samples	The study did not involve samples collected from the field.
Ethics oversight	All mice were bred and maintained in pathogen-free conditions and all procedures performed were approved by the Institutional Animal Care and Use Committee of Massachusetts General Hospital.

Note that full information on the approval of the study protocol must also be provided in the manuscript.

Flow Cytometry

Plots

Confirm that:

☒ The axis labels state the marker and fluorochrome used (e.g. CD4-FITC).

☒ The axis scales are clearly visible. Include numbers along axes only for bottom left plot of group (a 'group' is an analysis of identical markers).

☒ All plots are contour plots with outliers or pseudocolor plots.

☒ A numerical value for number of cells or percentage (with statistics) is provided.

Methodology

Sample preparation	The sample preparation and biological source of the cells is described in the manuscript.
Instrument	FACS Aria II (BD Biosciences)
Software	The flow cytometry data were analyzed by FlowJo 10 software
Cell population abundance	The purity of the samples was determined by rerunning flow cytometry (>90%).
Gating strategy	The gating strategies are described in the manuscript in Supplementary Figure 4d and Supplementary Figure 9

☒ Tick this box to confirm that a figure exemplifying the gating strategy is provided in the Supplementary Information.
